# *P. falciparum* Isolate-Specific Distinct Patterns of Induced Apoptosis in Pulmonary and Brain Endothelial Cells

**DOI:** 10.1371/journal.pone.0090692

**Published:** 2014-03-31

**Authors:** Nadine N'Dilimabaka, Zacharie Taoufiq, Sergine Zougbédé, Serge Bonnefoy, Audrey Lorthiois, Pierre Oliver Couraud, Angelita Rebollo, Georges Snounou, Dominique Mazier, Alicia Moreno Sabater

**Affiliations:** 1 Université Pierre et Marie Curie-Paris 6, UMRS 945, Paris, France; 2 Institut National de la Santé et de la Recherche Médicale, UMRS 945, Paris, France; 3 Institut Pasteur, Unité d'Immunologie Moléculaire des Parasites, CNRS URA 2581, Paris, France; 4 Institut National de la Santé et de la Recherche Médicale, U1016, Institut Cochin, Paris, France; 5 CNRS, UMR8104, Paris, France; 6 Université Paris Descartes, Sorbonne Paris Cité, Paris, France; 7 Laboratoire de Parasitologie-Mycologie, AP-HP, Hôpital Pitié Salpêtrière, Paris, France; University of Copenhagen and Rigshospitalet, Copenhagen, Denmark

## Abstract

The factors implicated in the transition from uncomplicated to severe clinical malaria such as pulmonary oedema and cerebral malaria remain unclear. It is known that alterations in vascular integrity due to endothelial cell (EC) activation and death occur during severe malaria. In this study, we assessed the ability of different *P. falciparum* clinical isolates to induce apoptosis in ECs derived from human lung and brain. We observed that induction of EC apoptosis was sensitive to the environmental pH and required direct contact between the parasite and the cell, though it was not correlated to the ability of the parasite to cytoadhere. Moreover, the extent of induced apoptosis in the two EC types varied with the isolate. Analysis of parasite genes transcript led us to propose that the activation of different pathways, such as *Plasmodium* apoptosis–linked pathogenicity factors (PALPF), PALPF-2, PALPF-5 and PF11_0521, could be implied in EC death. These observations provide an experimental framework to decipher the molecular mechanism implicated in the genesis of severe malaria.

## Introduction

The gamut of the clinical manifestations of malaria infections due to *P. falciparum* infection extends from asymptomatic to life threatening. Clinical severity encompasses a wide array of associated pathologies that include metabolic alterations, renal failure, liver and lung dysfunction, anaemia and cerebral malaria [1]

A characteristic feature of *P. falciparum* infection is the sequestration of parasitized red blood cells (PRBC) in various organs, such as the brain, lung, and placenta [2]. Sequestration results from the interaction between adhesive parasite-derived molecules expressed on the surface of the infected red blood cells and several receptors expressed on the surface of the vascular endothelium [3]. It has been noted from early post-mortem observations that the sequestered parasites might be unevenly distributed between the various internal organs, in a manner that appears to correlate with the type of severe pathology that led to death [4]. This notion has been supported by recent molecular analyses that showed differential *var* gene expression in the organs of patients who succumbed to malaria [5]. Cerebral malaria, the most studied and most dangerous of the severe manifestations, has consistently been associated with sequestration of *P. falciparum* to the brain vasculature [6], [7]. This is in agreement with recent indications that a subset of the PfEMP1 family mediates preferential cytoadhesion to the brain vasculature [8], [9], [10].

However, PRBC sequestration *per se* and the parasite's genotype are not sufficient to account for the diverse clinical manifestations in *falciparum* malaria [11], [12]. Sequestration is generally observed in all *P. falciparum* infections, yet progression to clinical severity is the exception rather than the rule. The importance of parasite sequestration to pathogenesis may be related to the downstream events induced in the host. Thus, it has been recently shown that sequestration causes considerable obstruction of blood flow [13], decreases tissue perfusion, the removal of parasite waste products, and generates hypoxia [14], [15]. A second consequence of parasite sequestration is the local release of bioactive toxins such as haemozoin, GPI anchors and histones [16], [17], [18]. This can induce recruitment of inflammatory mediators which could contribute significantly to the onset of severe malaria [19]. Based mainly on post-mortem and autopsy findings, it has become apparent that host cells, such as leucocytes or platelets, might also be sequestered in microvessels along with the PRBCs. These host cells might be involved in the pathogenesis of severe malaria, either through local effects on the microvessels or through distant effects mediated by the production of potentially deleterious mediators, such as pro-inflammatory cytokines, which can be detected in the circulation. A third consequence of parasite sequestration is the widespread activation of the endothelial cells (EC), which has been observed in mild as well as in fatal cases of malaria [20]. A procoagulant state has also been identified in these same patient populations, characterized by haemostatic alterations [21] thrombocytopenia [22] and microparticles production [23]. Finally, disruption of endothelial integrity is a pathological feature often associated with severe manifestations such as cerebral malaria [24] and pulmonary oedema [25].

Several mechanisms might account for the EC damage. Disruption of endothelial junction proteins and barrier permeabilization could result from cytoadherence-induced signalling [26], [27] through formation of endothelial cup like structure at the site of PRBC adhesion [28]. Additionally, cytoadherence-independent mechanisms, such as metabolic acidosis due to PRBC maturation [29] or the release of parasite factors [30] such as merozoite proteins [31], histones [16] and plasma uric acid [32] also contribute to endothelial damage. Finally, the induction of EC apoptosis by PRBC, platelets and neutrophils would severely disrupt endothelial integrity [26], [33], [34], [35], [36]. Finally, it was shown that the potential of *P. falciparum* to cause human lung EC (HLEC) apoptosis *in vitro* varies with the isolate [37], [38], and that this might in turn be linked to the expression of a subset of parasite genes named “*Plasmodium* apoptosis–linked pathogenicity factors” (PALPFs) [39]. Recent studies have confirmed that the parasite-induced apoptosis described *in vitro* also occurs in the brain, lung and kidney of fatal malaria cases [33], [40]

While different mechanisms for the EC damage have been studied, its role in the gamut of the clinical manifestations remains poorly understood. In this study, we wished to ascertain whether the apoptotic potential of PRBC is the same for ECs of different origin, and whether this varies for distinct *P. falciparum* isolates. To this end, we used HLEC [41] and the human brain microvascular EC (HBEC) line, hCMEC/D3, that is used for pathogenic and drug transport studies [42], to assess the ability of three parasite strains derived from clinical isolates to induce apoptosis in these brain or lung ECs.

## Materials and Methods

### EC culture

HLECs were a gift from Dr. F. Gay, (UMRS 945). HLECs were cultured in a lung culture medium (LCm) composed of M199 medium (Invitrogen) supplemented with 5 µg/mL of EC growth factor (Millipore) and 10% of heat inactivated Foetal Bovine Serum (FBS) (Gibco) as previously described [41]. HLECs from the ninth to the thirteenth passages were used for experiments.

The HBEC line hCMEC/D3 was a gift from Prof. P. O. Couraud (INSERM, U1016, Paris, France). This cell line shows a stable normal karyotype, maintained contact-inhibited monolayers in tissue culture, exhibited robust proliferation in response to endothelial growth factors, and formed capillary tubes in matrix but no colonies in soft agar. The hCMEC/D3 cells expresse telomerase and grow indefinitely without phenotypic dedifferentiation. These cells expressed chemokine receptors, up-regulated adhesion molecules in response to inflammatory cytokines, and demonstrated blood-brain barrier characteristics, including tight junction proteins and the capacity to actively exclude drugs [43]. HBECs were cultured in a brain culture medium (BCm) composed of EBM-2 basal medium (Lonza) supplemented with growth medium Bullet kit 2 (Lonza) as previously described [43]. HBECs from the twenty-ninth to the thirty-sixth passages were used for the experiments. Both cell lines were mycoplasma free as assessed by PCR kit (Minerva Biolabs).

### *P. falciparum* clinical isolates

Clinical isolates of African origin were kindly supplied by Dr. P. Buffet (UMRS 945) and Dr. M.-N. Ungeheuer (ICAReB platform, Clinical Investigation and Research Bioresources, Institut Pasteur, France). The isolates were obtained during routine care and after informed consent of patients (Globex program, Cochin Hospital Human Ethics Committee) admitted to different French hospitals (Necker, La Pitié-Salpêtrière, Bichat). The protocol was approved by the Human Ethics Committee CPP Ile-de-France III (26 March 2004, under ref. 321_040402_C). Patient data were anonymized prior to the experimental work. The three isolates were deliberately obtained from selected patients presenting with different clinical symptoms. Isolate A was obtained form a patient who presented with a 4.5% *P. falciparum* parasitaemia and a fever of 38.5°C. Isolate B was obtained from a patient who presented with a 6.4% *P. falciparum* parasitaemia and a fever of 39.2°C who also suffered from slumber and asthenia. Isolate C was obtained from a patient who presented with a 14% *P. falciparum* parasitaemia, a fever of 39°C, sweats, body aches, malaise, asthenia and was unconscious during examination. Patient A and B were considered to have acute malaria, whereas patient C was considered to suffer from severe malaria.

The isolates were adapted to *in vitro* culture and were confirmed to be mycoplasma-free by PCR, prior to cryopreservation of several aliquots. For each assay, one of the aliquots was thawed and the parasites were cultured for a maximum of 6 weeks as previously described [29]. Mature PRBCs (late trophozoites and schizonts) were obtained by the gelatine floating method [44].

### EC apoptosis assay

HLECs and HBECs were grown until confluence in 24-well plates (Costar). For each experiment, mature PRBCs forms were adjusted to a 50% parasitaemia, 1% haematocrit and co-cultured with HLECs and HBECs in parallel for 24 h in LCm or BCm, respectively. EC apoptosis was measured by annexin V/propidium iodide (PI) staining following the manufacturer's recommendation (Miltenyi Biotec). The ECs were washed three times with their respective culture media, once more with ice-cold binding buffer and stained with annexin V for 15 minutes in the dark. After washing, the ECs were stained with PI. Apoptosis was quantified by direct microscopy and analytical digital photomicroscopy. For each well, healthy and apoptotic ECs were discriminated by annexin V/PI staining. Concurrently, homogeneously distributed fields (magnification ×200) were imaged using a Leica epifluorescence microscope (Leica DMI 4000B, Leica Microsystems, Wetzlar, Germany) with the red filter for PI staining and the FITC filter for annexin V staining, and the images recorded with a Zeiss camera (AxioCam MRc5, Oberkochen, Germany). The number of annexin V/PI stained cells per 1000 ECs was then counted by microscopy and image analysis and the percentage was calculated.

### Influence of culture medium on EC apoptosis

Co-cultures of isolates with HLECs or HBECs were carried out in parallel using the LCm and BCm respectively or two modified media that lacked bicarbonate and had reduced buffer efficiency [29]. For HLECs, the modified medium contained RPMI 1640 supplemented with 10% FBS and ECGF, and was called MLCm. For brain ECs, it contained RPMI 1640 supplemented with the growth medium Bullet kit 2 (Lonza), and was called MBCm. Co-cultures were carried out in the same conditions of parasitaemia and haematocrit described above. At the end of this period, the supernatants were collected and their pH determined using a pH meter (Radiometer, Copenhagen). AnnexinV/PI staining was performed on the HBECs and HLECs to quantify apoptosis after PRBC removal and washing.

### PRBC adhesion assay

The level of cytoadhesion of clinical isolates to EC was determined as previously described [29]. Briefly, HLECs and HBECs were expanded in their respective culture medium in 8-well Lab-Tek™ chamber slides (Nunc) until 80% confluence and stimulated with 150 U/mL of tumor necrosis factor-α (TNFα) (Biosource) for 24 hours. At the end of this period, the ECs were fixed with 4% paraformaldehyde for 15 minutes and kept at 4°C until use. Mature PRBCs were co-cultured with TNFα-stimulated HLEC and HBEC at 50% parasitaemia and 1% haematocrit for 1 hour at 37°C. During co-incubation, gentle agitation was performed every 15 minutes. At the end of incubation, the Lab-Tek wells were removed and the cultures were washed in RPMI 1640 medium to remove unfixed PRBCs. The slides were gently washed up and down in the medium, fixed in 2% glutaraldehyde, and stained with Giemsa (CML). The number of adherent PRBCs per 500 HLECs or HBECs was determined by microscopy.

### EC apoptosis induced by panned isolates and trypsinized PRBCs

The level of cytoadherence two isolates (A and C) was increased using the panning method as previously described [29]. Briefly, mature PRBCs from each isolate were co-cultured with HLECs and HBECs at 5% hematocrit for 1 hour at 37°C with gentle agitation every 15 minutes. Non-adherent parasites were removed by washing with RPMI 1640 medium. Adherent parasites were harvested by gently pipetting complete parasite culture medium on the cells to facilitate PRBC detachment. The parasites thus obtained were cultured to increase parasitaemia. In total three cycles of panning were carried out.

To evaluate the role of cytoadhesion, the mature PRBCs were trypsin treated to reduce adhesion to HLECs and HBECs [45]. The parasites enriched by gelatine floatation were washed in Hank's balanced salt buffer and incubated either in buffer or in 0.5 mg/mL trypsin/EDTA at a 10% final hematocrit for 5 minutes at room temperature. The reaction was stopped by adding RPMI 1640 supplemented with 5% foetal bovine serum. Trypsinized PRBCs were stained with Giemsa before and after trypsinisation to confirm that PRBC integrity was maintained.

The ability of the panned control or trypsinized PRBCs to induce HLEC or HBEC apoptosis was evaluated using the co-culture conditions with the normal and the modified media described above. Apoptosis quantification was carried out after 24 h of incubation.

### PRBC supernatants and transwell assays

In order to evaluate the impact of parasite soluble factors, mature PRBCs were cultured in 24-well plates (Costar) at 50% parasitaemia at 1% haematocrit in parallel using the MLCm or the MBCm. The supernatants from these cultures were then recovered by centrifugation (5 min, 2000 g) and added for 24 hours to HLECs and HBECs that were grown to confluence in 24-well plates. The apoptosis quantification was carried out at the end of this period.

We used transwell inserts, polyester, 0.4-µm pore size (Corning Life Sciences) for the non-contact assay. HLECs and HBECs were grown until confluence in the lower compartment of a 24-well plate (Costar). Transwell inserts were added to each well and PRBCs at 50% parasitaemia and 1% haematocrit were co-cultured in the upper compartment for 24 h before apoptosis quantification.

### RNA extraction and reverse transcription (RT)-PCR

Mature PRBCs were co-cultured with HLECs and HBECs at 20% parasitaemia and 5% haematocrit during 4 hours at 37°C and 5% CO_2_. The PRBCs and the ECs were lysed and the RNA was purified using the PureLink RNA Mini Kit (Ambion) according to the manufacturer's recommendations. RT was carried out using SuperScript VILO cDNA Synthesis Kit (Invitrogen). PCR amplifications were carried out with the SYBR Green method (Invitrogen), using the primers (Table 1) previously described [39] at 200 nM final concentration, 95°C 4 min, then 95°C 30 s, 60°C 30 s, 72°C 1 min for 30 cycles and then 72°C 1 min. Gene expression data from seven selected genes were normalized to the parasite's 18S ribosomal genes' cDNA level. These values were used to calculate variations in expression between cultures carried out under standard conditions or under conditions that enhance EC apoptosis. Variations in expression were expressed as a ratio of the values determined for parasites co-cultured using modified culture media to those obtained for parasites co-cultured using the standard culture media.

**Table 1:** Primers used for reverse-transcriptase polymerase chain reaction (RT-qPCR) experiments.

Genes		Real time PCR primers	Size (bp)
**18s**	Forward	CTTTTGAGAGGTTTTGTTACTTTGAGTAA	29
	Reverse	TTCCATGCTGTAGTATTCAAACACAA	26
**PALPF-2**	Forward	CGTCTGTGTCATATGCTCATT	21
	Reverse	TGAGGGCAAACTCTCTTCAT	20
**PALPF-3**	Forward	AAGAACCCACAGAATAATTTCTG	23
	Reverse	TTGGTTTTGCTTTGTTCCA	19
**PALPF-4**	Forward	TATTTTTCACACCATCCTTGTTACAA	26
	Reverse	AAGTCGATGATCCAGTTATAATAACATTG	29
**PALPF-5**	Forward	AAGATACGTTGCATCTCAACCTT	23
	Reverse	TGCTGGTCTTAATGTCCATCTTT	23
**PFL1955W**	Forward	GAAAGCTATCACGTGCGACGCTAA	24
	Reverse	CCATTCCTCGAACCAGCGAAGAT	23
**PF11_0521**	Forward	GGGACGATTGGTGGAATGCGAATA	24
	Reverse	CTTCTGCCCATTCCTCAAACCATCTTAAA	29
**MAL7P1.81**	Forward	GAGAGTTTCAGACGGGACACAAATAGG	27
	Reverse	AAATTCGACAAATCTCACCGGACCAT	26

### Statistical analysis

Mean and standard deviations have been determined after 3 independent experiments. An analysis of variance (ANOVA) followed by post hoc analysis with Tukey test was used to establish the levels of significance: * P≤0.05, **P≤0.005, ***P≤0.0005.

## Results

### Distinct parasite isolates induce different patterns of HLEC and HBEC apoptosis

**Figure 1:**
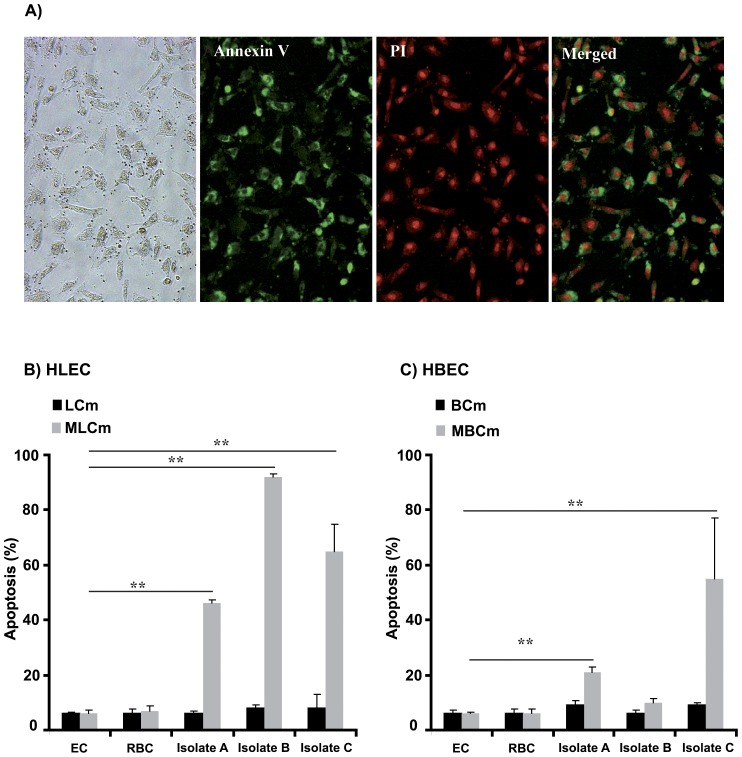
The induction of apoptosis in HLECs or HBECs co-cultured with *P. falciparum* PRBCs depends on the culture conditions. A) Co-cultured endothelial cells (ECs) and parasitized red blood cells (PRBCs) after Annexin V and propidium iodide (PI) staining. B) Human lung endothelial cell (HLEC) apoptosis induced by the different *P. falciparum* isolates (A, B and C) when the co-cultures are made in the standard culture media (LCm) or in the modified culture medium (MLCm). C) Human brain endothelial cell (HBEC) apoptosis induced by the different *P. falciparum* isolates (A, B and C) when the co-cultures are made in the standard culture media (BCm) or in the modified culture medium (MBCm). The percentage of apoptotic cells was calculated after microscopy enumeration of Annexin V and propidium iodide positive cells in 1000 ECs. The results are expressed as mean values, and standard deviation are shown for duplicate wells from three biological replicates. Significance at *p*<0.005 is indicated by **.

We analyzed the potential of three *P. falciparum* isolates from distinct clinical isolates (A, B and C) to induce the apoptosis of human ECs derived from the lung or the brain using annexin V/PI staining. The use flow cytometry to measure the level of apoptosis in the ECs proved problematic. Indeed, we had previously shown that PRBCs maintained under acidified conditions are induced to undergo an apoptosis-like cell death [29] and consequently these PRBCs stained with annexin V/PI. Thus, in the present study, where acidification occurs in the co-cultures, the presence of annexin V/PI-stained PRBCs obscured the measurement of EC apoptosis by flow cytometry analysis of ECs and PRBCs dissociated from the co-cultures. We therefore, assessed EC apoptosis by annexin V/PI staining of the adherent ECs. Healthy and apoptotic ECs were easily discriminated by annexin V/PI staining as shown in Figure 1A. In cultures where the recommended media for HLEC or HBEC culture were used (LCm or BCm, respectively) only low levels of apoptosis (10%) were observed in the presence of PRBCs. These values did not differ from the basal levels observed when the ECs were cultured alone or in the presence of uninfected red blood cells (Figure 1B and 1C). By contrast, significant levels of apoptotic ECs were observed for some of the co-cultures when the modified culture media with limited buffering capacity (MLCm and MBCm) were used for cultivation (Figure 1B and 1C).

In co-cultures maintained under conditions that are permissive to the PRBC-induced EC apoptosis, it was interesting to note that the apoptotic potential not only differed for the *P. falciparum* isolate used, but that it also varied with the type of ECs present in the culture (Figure 1B and 1C). Isolate A caused moderate levels of apoptosis in HLECs whereas HBECs were relatively less affected. Isolate C induced high levels of apoptosis in both EC lines. By contrast few of the HLECs co-cultured with isolate B survived while low apoptosis was observed in co-cultures with HBECs.

### Culture medium pH decrease can potentiate the proapoptotic effect of PRBC on EC

**Figure 2:**
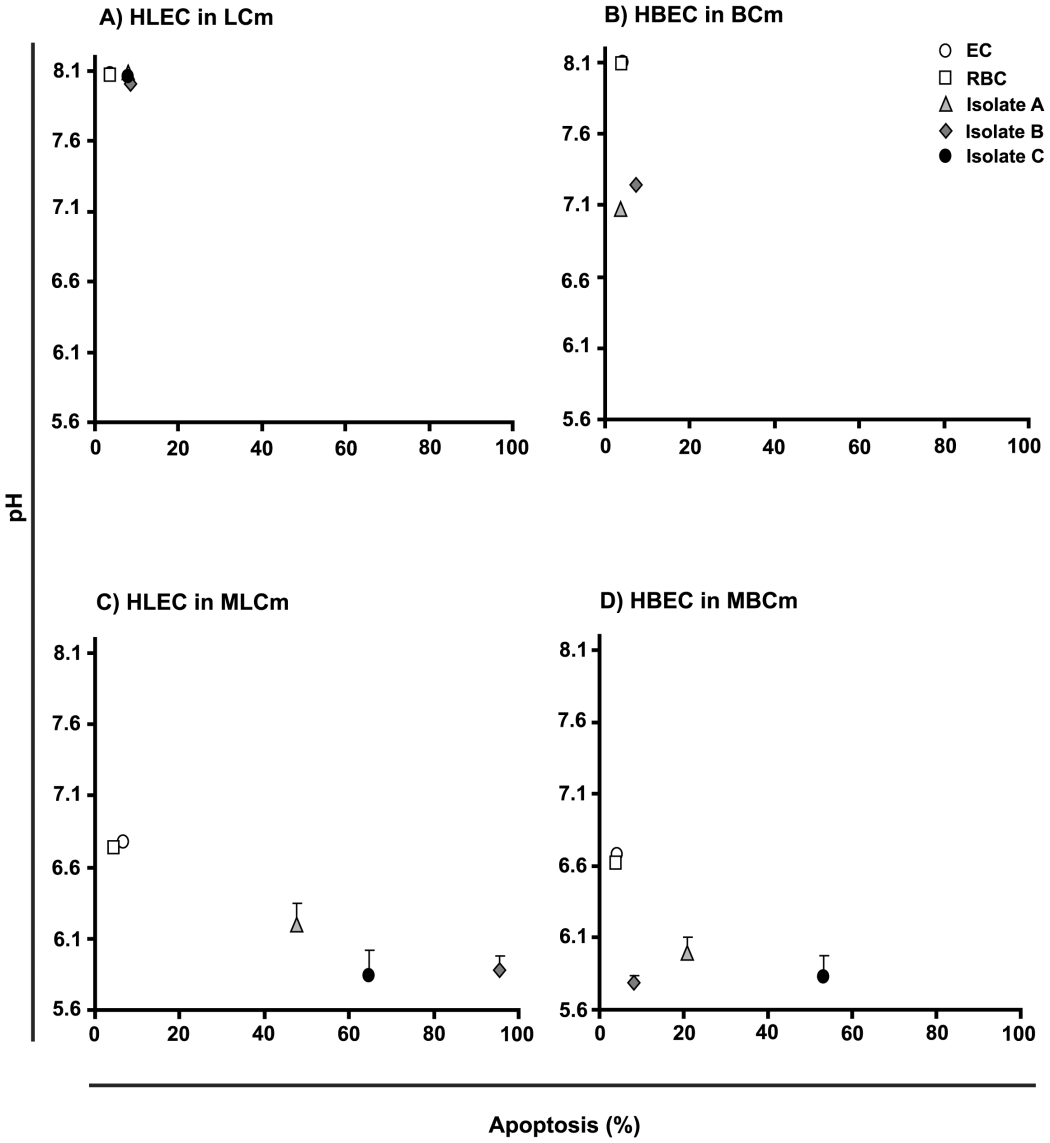
The effect of the medium's pH on EC death in co-cultures with *P. falciparum* PRBC. (A) Correlation between the medium's pH at the end of incubation and the levels of induced apoptosis in HLEC (A) and HBEC (B) co-cultured with the isolates in the standard media, and in the modified media (C) and (D), respectively. The pH values are expressed as means with standard deviation from duplicate wells from three biological replicates.

We measured the pH 24 h after the initiation of the co-cultures and correlated it with the observed levels of apoptotic ECs. The LCm displayed a high buffering capacity in that the pH did not vary in any of the experimental co-cultures or controls (Figure 2A). The BCm was less efficient, such that a drop of approximately 1 pH unit was noted in co-cultures with PRBCs, but none in the controls (Figure 2B). The modified media (MLCm and MBCm) had, as expected, a substantially reduced buffering capacity. In simple cultures or in the presence of uninfected red blood cells, the pH was close to 6.6 after 24 hours and it fell to lower levels (6.1 and less) when the ECs were co-cultured with *P. falciparum* PRBCs (Figure 2C and 2D). When the pH values were correlated with the percentage of apoptotic HLECs, we observed an increase in the percentage of apoptotic HLECs co-cultured with the different isolates under conditions that led to a decrease in the pH (Figure 2C). However, although the resulting low pH values were similar for the three isolates, the percentages of the induced HLEC apoptosis were different for each isolate, with isolate B inducing the highest levels. This tendency was less evident when the lower pH values, also similar for the three isolates, were correlated with the percentage of apoptotic HBECs. An increase in this percentage was only noted for isolate C. These results showed that culture medium pH decrease can potentiate the proapoptotic effect of PRBCs on ECs, but the differences in the levels of apoptosis induced in the two EC lines by the three parasite isolates led us to propose that isolate-specific factors play a role in the apoptotic death of ECs.

### Induction of EC apoptosis depends on contact with PRBCs but not on their cytoadherence profile

**Figure 3:**
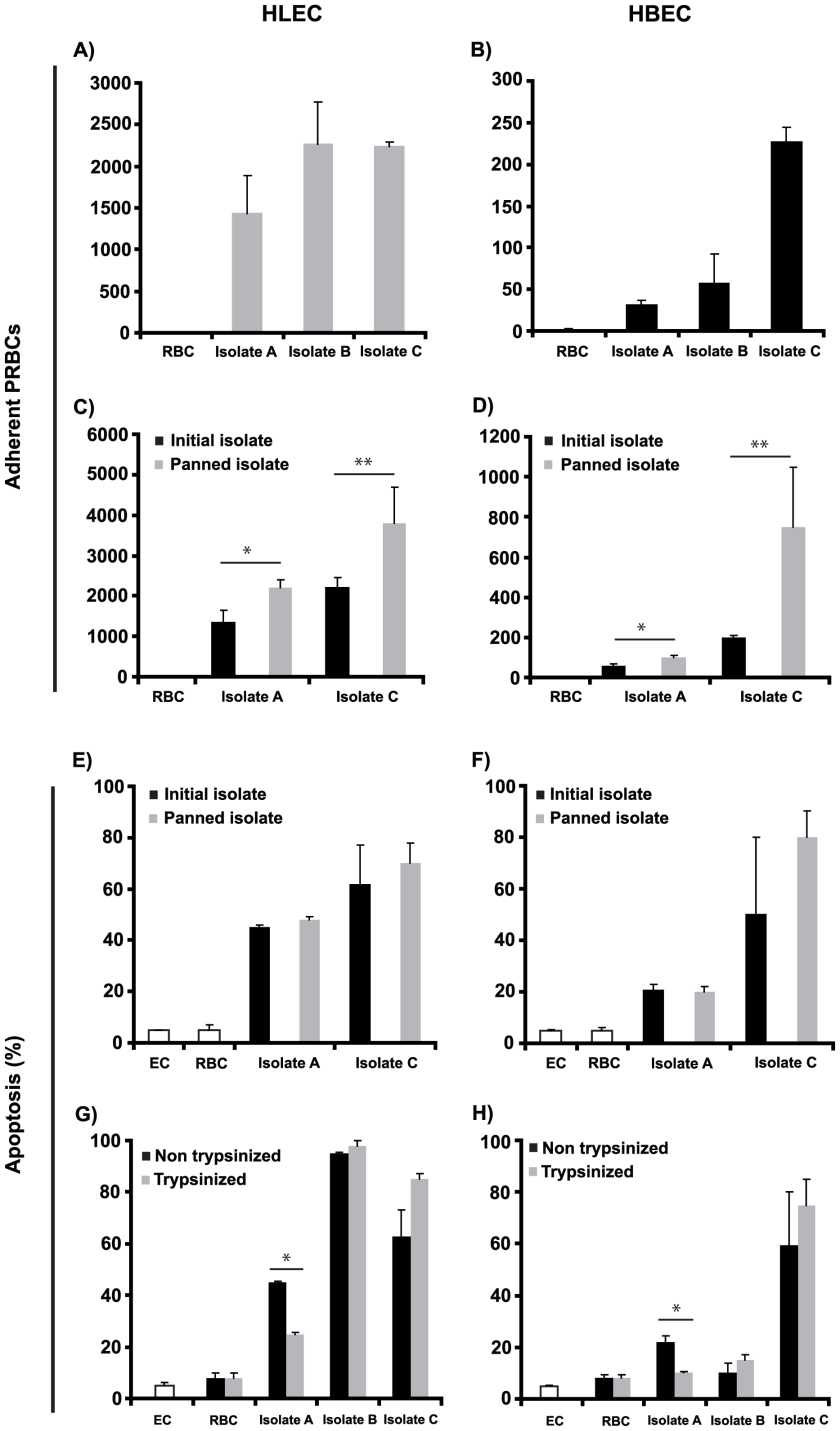
Patterns of PRBCs cytoadherence in relation to HLEC and HBEC apoptosis. The number of adherent PRBC per 500 ECs from each isolate to HLEC (A) and HBEC (B), as enumerated after incubation for 1 h and three washed to remove non-adherent cells. Isolates A and C with increased levels of cytoadherence selected for by panning on HLEC and HBEC, (C) and (D) respectively, do not lead to increased apoptosis when co-cultured with HLEC and HBEC, (E) and (F) respectively. Trypsin treatment that abolishes cytoadherence in the isolates does not alter their ability to induce cell death of co-cultured HLEC and HBEC (G) and (H) respectively. In all panels, the results are expressed as means with standard deviations from duplicate wells from three biological replicates. Significance levels are denoted by * for p<0.05, and ** p<0.005.

Given the acknowledged role of cytoadherence in the pathogenesis of *P. falciparum* infections, we wished to determine whether the patterns of EC apoptosis correlated with distinct levels of cytoadherence of the different isolates to the cultured ECs. All three isolates displayed strong cytoadherence to HLECs (Figure 3A). Cytoadhesion to HBECs was much less pronounced, though the levels of cytoadherence observed for isolate C were four-fold higher than those for isolates A and B (Figure 3B). Although the levels of cytoadhesion observed did not directly mirror the extent of apoptosis induced in the ECs, the two appeared to correlate, suggesting a relationship between cytoadherence and induction of apoptosis.

If this were to be the case, then the apoptogenic potential of a given isolate should be enhanced if its level of cytoadherence were increased. Thus, we selected two of the isolates (A and C) and selected them for higher levels of cytoadherence through 3 panning rounds on HLECs and HBECs (Figure 3C and 3D). However, the original parasites and those selected for higher cytoadherence did not differ in their ability to induce cells apoptosis in the co-cultures with HLECs and HBECs (Figure 3E and 3F). These observations indicated that the apoptogenic potential of the parasite was not linked to their cytoadhesion profile. This conclusion was confirmed when trypsin treatment of the parasites, which abolishes their ability to cytoadhere, did not alter the levels of induced cell apoptosis in isolates B and C, though a minor but significant reduction was observed for isolate A (Figure 3G and 3H).

**Figure 4:**
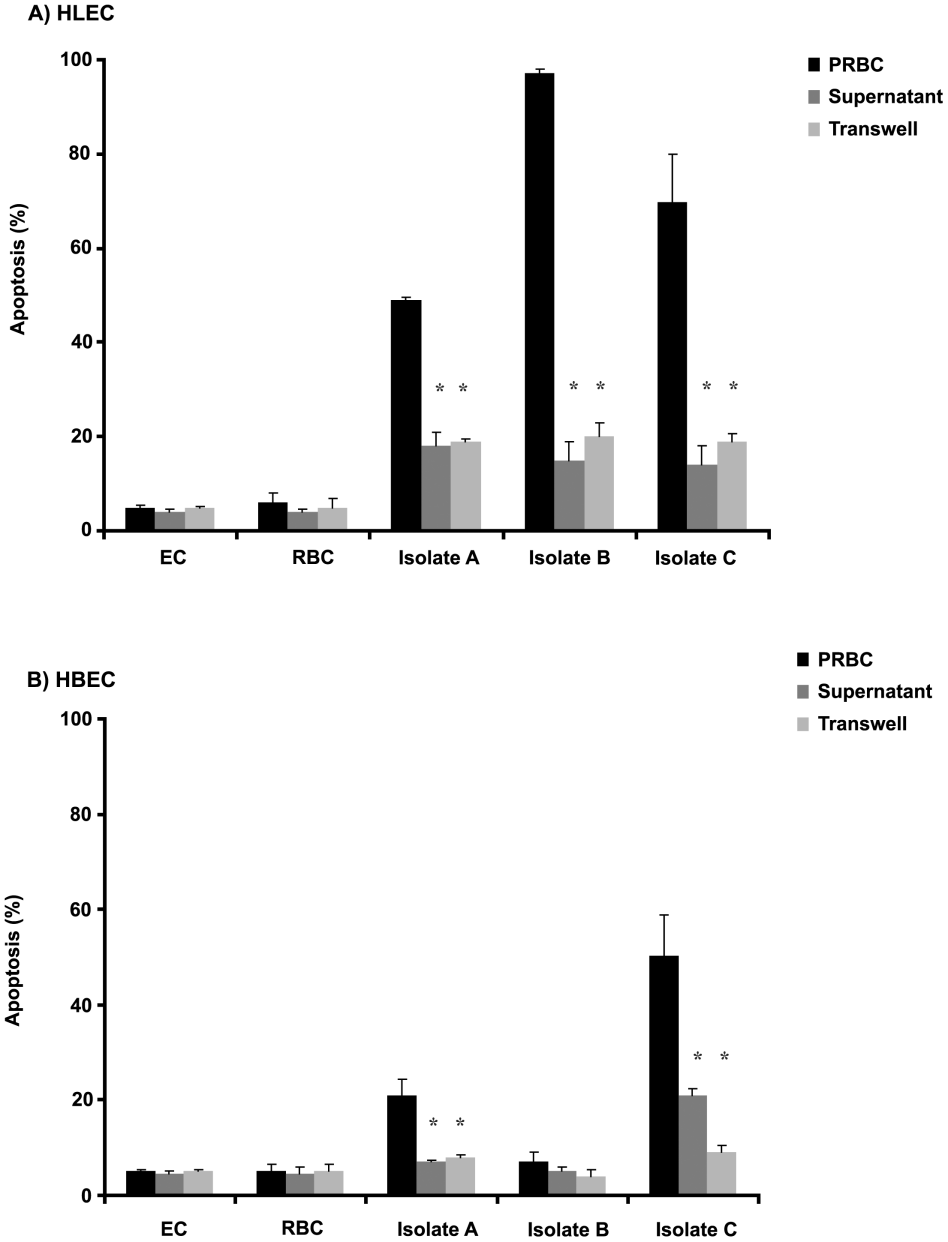
Direct cell-to-cell contact is needed for optimal induction of EC apoptosis. HLEC (A) and HBEC (B) co-cultured with individual supernatants harvested from cultured *P. falciparum* isolates or in transwell plates where the PRBC are incubated on top of the ECs. The results show reduced levels of apoptosis as compared to those observed in co-cultures where PRBC and ECs are in direct contact. For all experiments the means and standard deviation were calculated from duplicate wells from three biological replicates. Significance at *p*<0.05 is indicated by *.

Nonetheless, when direct contact between the PRBCs and the cultures of ECs was prevented, either through the use of transwell cultivation, where the two cell types were physically separated by a porous membrane, or by incubation of HLECs and HBECs with PRBC culture supernatant, substantially lower levels of induced cell apoptosis could be observed in the cultured ECs (Figure 4A and 4B).

### Associating parasite gene expression with apoptotic potential

The induction of differential levels of cell apoptosis in two types of ECs under one culture condition but not another, suggests that in these environmental conditions, parasites might also differ in the expression of one or more proteins implicated in EC apoptosis. We analyzed a random selection genes previously found to be linked with parasite apoptogenicity [39] in isolate B and isolate C co-cultured with HLECs or HBECs in standard medium or in the modified medium (Table 2). We also analyzed a set of genes showing preferential transcription *in vivo* in isolate C (MAL7P1.81/eukaryotic translation initiation factor) or PF11_0521 and PFL1955w *var* genes in isolate B (S. Bonnefoy, unpublished results).

**Table 2:** Variation of gene expression when *P. falciparum* isolates were incubated with ECs.

Genes	HLEC		HBEC	
	Isolate B	Isolate C	Isolate B	Isolate C
**PALPF2**	NS	NS	NS	1.66±0.21*
**PALPF3**	NS	NS	1.73±0.08**	NS
**PALPF4**	NS	NS	1.66±0.16*	1.37±0.12*
**PALPF5**	2.2±0.16***	1.27±0.003***	NS	NS
**MAL7P1.81**	NS	NS	1.97±0.22*	2.03±0.02***
**PF11_0521**	NS	NS	NS	1.47±0.003***
**PFL1955w**	NS	NS	2.8±0.27*	NS

The implication of the seven selected genes in the ability of isolates B and C to induce the lung and brain EC death was represented as a ratio by comparing their RNA levels in co-cultures using the modified culture mediums, where EC apoptosis death was detected. For all experiments the means and standard deviation were calculated from duplicate wells from two biological replicates.

*p<0.05,

**p<0.005,

***p<0.0005.

NS = Non Significant.

In co-cultures under conditions that are permissive to the PRBC-induced EC apoptosis, the parasite's transcriptome is significantly altered, but it was interesting to note that the expression patterns for the selected genes differed between the parasites co-cultured with HLEC and those co-cultured with HBEC. Thus, one gene only, PALF-5, was significantly up regulated in both parasite isolates able to induce HLEC cell apoptosis. More complex results were obtained when isolates B and C were co-cultured with HBEC: PALF2 and PF11_0521 were only increased in isolate C, able to induce HBEC apoptosis, as compared to isolate B pointing to a possible involvement of these genes in brain EC apoptosis.

## Discussion

Clinically, the course of the *P. falciparum* infection varies from the chronic asymptomatic to the rapidly fatal, and the gamut of symptoms extends from non-specific mild fevers to clearly delineated severe organ-specific syndromes. Whereas it is not possible to predict the clinical evolution at the level of the individual, the risk for a given population to develop a particular pathology is generally known (for example, severe anaemia or cerebral malaria in adults *versus* children in specific endemic settings) [46]. It is accepted that the hosts' genetic background, their level of acquired immunity, the exposure to which they are subjected, and other factors such as nutritional and general health status influence the expression of clinical severity. In *P. falciparum* infections, the extreme levels of sequestration amplify the interactions between the parasite and the endothelium and exacerbate inflammatory responses and endothelial activation. Thus, for infections with this parasite species alterations in the function and in the integrity of the endothelial barrier are likely to play a key role in the development of clinical severity. In here we focused on the induction of EC apoptosis by interactions with the PRBC, a phenomenon that is most likely to impair endothelial integrity.

We first confirmed previous observations [29] indicating that the induction of EC death, which would lead to the disruption of endothelial integrity, is potentiated by the acidification of the culture medium, most probably due to lactic acid production by the parasite. This is consistent with the established association of acidosis with poor clinical outcomes [47]. Such a potentiating effect has been described for TNF, a cytokine whose plasma levels are elevated in severe malaria. Thus, TNF-stimulation of EC was shown to potentiate the proapoptotic effect of platelets on ECs [36], [48], which led to hypothesize that PRBC-induced EC apoptosis is exacerbated in ECs that have been previously exposed to platelets. These results suggest that events at the EC microenvironment could play an important role in triggering EC apoptosis. We are aware that observation of both annexin-V and PI staining in experiments presented here could be interpreted as evidence of necrosis rather than of the later stages of apoptosis. In our case we consider it most likely that the double staining is indicative of late apoptosis because we had previously demonstrated that exposure of the ECs to PRBCs for 6 hours [34] or 24 hours [26] induces their apoptosis as detected by annexin-V staining, and that this induction was prevented by treatment with caspase inhibitors. In the present set experiments where exposure to the apoptotic agent was 24 hours, the cells would have reached the later stages of apoptosis when, cell membrane integrity would be lost, thus accounting for the staining with annexin-V and PI.

The interest of the observations presented here lies in the following: Whereas the three distinct isolate used led to a similar decrease in the pH, the levels of apoptosis induced varied substantially not only for each of these isolates but also for the lung and the brain ECs tested. The differences in the levels of apoptosis observed between the two EC lines in response to each of the parasite isolates could be related to the structural and functional heterogeneity known to distinguish ECs from different organs [49], or to the fact that the HBEC line used here (hCMEC/D3) are derived from an immortalized cell line whereas the HLECs are primary cells. The robustness of the results obtained using hCMEC/D3 cells have been proved in pathogenic as well as in drug transport studies [42]. Thus, although differences could exist between a primary and an immortalized cell, in our case these differences are minor. We could not distinguish between these two possibilities because primary human brain ECs were not available to us. Nonetheless, the results indicate that for each of the EC lines used, the induction of apoptosis was dependent on the parasite's genotype.

Given the primary role of parasite sequestration to the microvasculature in leading to vital organ dysfunction [50] and the preferential binding of parasites to some organs in patients dying of *falciparum* malaria [5], we first expected that the induction of EC apoptosis would be correlated with the levels of cytoadhesion of the three *P. falciparum* isolates to the two types of ECs tested. However, the data from the trypsin-treated PRBCs, which cytoadhere poorly, clearly indicated that EC apoptosis was independent of cytoadhesion *per se.* We then showed that EC apoptosis necessitated direct contact between the PRBC and the ECs. Similar cytoadhesion-independent effects on the endothelium were recorded in previous studies, for e.g. PRBC-induced ICAM-1 up regulation in EC or PRBC-induced endothelial permeability [29], [30], [51]. In these cases, cytoadherence would play an indirect role by increasing the levels of EC-PRBC contact as well as the local parasitaemia, thus enhancing the effects of factors released from the maturing parasites on the endothelium.

The dependence on direct contact led us to propose that trypsin-resistant membrane components or factors released by the maturing parasite or from the bursting mature schizont, such as lactic acid, glycosylphosphatidylinositol (GPI), haemozoin, uric acid, and histones or from factors derived from oxidative stress [16], [18], [32], [52], [53], induce EC apoptosis. Irrespective of their nature, the apoptogenicity of any soluble factors alone depends on the acidification of the environment, since the induction of apoptosis was not pronounced in co-cultures incubated in culture medium with high buffering capacity. Moreover, we had shown in a previous study that under acidic culture conditions PRBCs are induced to undergo apoptosis [29]. Thus, we cannot dismiss the possibility that factors released from apoptotic PRBCs would also contribute to apoptosis observed in the ECs. At present, the nature of these factors remains a matter for future investigation. From our observation we predict that their production or structure would differ between distinct parasite isolates.

PALPFs transcript in several *P. falciparum* isolates has allowed differentiate its ability to induce EC apoptosis [39]. In our study, when parasite transcript was compared between an EC apoptotic and non apoptotic environment, the result obtained pointed to two PALPFs, described as hypothetical transmembrane proteins: PALF-5, PALF 2. In addition, PF11_0521, a gene for a transmembrane protein belonging to the PfEMP1 *var* gene family, has also been up regulated in these apoptotic conditions. Variation of the transcription profile of *var* genes must be interpreted carefully since it can change during clinical isolates adaptation to *in vitro* culture [54]. In this study, we show another level of complexity in the analysis of transcription profile of this *var* gene, since it can also change and be up regulated when PRBCs are in a slight acidic environment and in contact with the ECs. Therefore, this study highlights a new aspect in the interaction between the PRBCs and ECs: the environment plays a crucial role in modulating the expression of parasite genes potentially implicated in the EC death.

We are aware that the observations presented here derive from three distinct *P. falciparum* isolates only. Nonetheless, the strikingly distinct patterns of EC death induction are sufficient to formulate a hypothetical framework to account, at least in part, for the complex nature of pathogenesis in malaria, and that would be consistent with long-standing clinical observations and current hypotheses. The notion that *P. falciparum* strains differ in pathogenicity has found support from epidemiological and experimental observations [55]. Many factors were proposed to account for these differences: variations in multiplication rate, in antigenicity, and in the production and nature of an as yet hypothetical malaria toxin. The discovery of a multigene family of proteins that mediate cytoadherence (PfEMP1) to distinct endothelial receptors provided a plausible hypothesis whereby the type and severity of the symptoms that might result from infection by a particular isolate would depend on the immune selection of subpopulations expressing distinct PfEMP1 proteins which would in turn modify the extent of sequestration to different organs.

Our observations suggest another layer of complexity. Each isolate would have a distinct propensity to cause EC apoptosis and subsequent alteration of the endothelial integrity, the extent of which would vary with the rise of local acidosis and possibly the particular properties of the EC in the different organs. In this manner, the clinical severity consequent to the disruption of vascular endothelial integrity of a particular organ would only become manifest when the parasites that preferentially sequester in this organ happen to be also those that induce substantial apoptosis to its ECs. Parasite sequestration, which usually occurs in regions of relative slow blood flow, will ensure a lengthy and direct contact between the PRBC and the endothelium. It will also lead to an increase in the local concentrations of released parasite factors, to a decrease in the local pH, and to activation of the EC and the inflammatory responses. These alterations of the EC microenvironment would in turn affect the parasite itself.

The very high levels of apoptosis induced in the ECs cultured *in vitro* under acidic conditions are unlikely to reflect the general situation *in vivo*. However, a recent histological study conducted on post-mortem brain necropsies showed levels of apoptosis approaching 80% for brain ECs [40]. It is likely that such levels are not reached in patients who survive the infection or who present with less severe malaria. In these patients, it is likely that counterbalancing protective host responses are operational. These would include effective maintenance of physiological pH levels, host immune responses such as macrophages clearing released parasite material or antibodies that would neutralise any apoptogenic soluble factors, as observed for GPI in an animal model [56]. Furthermore, plasma proteins such as activated protein C has been described to afford some protection to vascular ECs from apoptosis [57]. Finally PRBC-induced EC apoptosis is triggered/regulated by the NF-κB transcription factor [35], which in turn is implicated in the trigger and regulation of many anti-apoptotic pathways.

In conclusion, the results presented here provide a new proposition to explain the complex syndrome of severe malaria. Further studies should be carried out with a large number of isolates corresponding to various clinical conditions in order to confirm the implication of this mechanism in the pathological process that leads to lung or brain lesions. This could lead to developing strategies to mitigate the damage to various organs and improve prognosis.
